# Risk Factors for Vitamin D Deficiency among HIV-Infected and Uninfected Injection Drug Users

**DOI:** 10.1371/journal.pone.0095802

**Published:** 2014-04-22

**Authors:** Allison A. Lambert, M. Bradley Drummond, Shruti H. Mehta, Todd T. Brown, Gregory M. Lucas, Gregory D. Kirk, Michelle M. Estrella

**Affiliations:** 1 Department of Medicine, Division of Pulmonary and Critical Care, Johns Hopkins University, Baltimore, Maryland, United States of America; 2 Department of Epidemiology, Johns Hopkins University, Baltimore, Maryland, United States of America; 3 Department of Medicine, Division of Endocrinology and Metabolism, Johns Hopkins University, Baltimore, Maryland, United States of America; 4 Department of Medicine, Division of Infectious Diseases, Johns Hopkins University, Baltimore, Maryland, United States of America; 5 Department of Medicine, Division of Nephrology, Johns Hopkins University, Baltimore, Maryland, United States of America; University of Sydney, Australia

## Abstract

**Introduction:**

Vitamin D deficiency is highly prevalent and is associated with bone disease, cardiovascular disease, metabolic syndrome and malignancy. Injection drug users (IDUs), with or without HIV infection, are at risk for these conditions; however, limited data on vitamin D deficiency exist in this population. We determined the prevalence and correlates of vitamin D deficiency among urban IDUs in the AIDS Linked to the IntraVenous Experience (ALIVE) Study cohort.

**Methods:**

For this cross-sectional sub-study, vitamin D deficiency was defined as a serum 25(OH)-vitamin D level <20 ng/mL. Multivariable logistic regression was used to identify factors independently associated with vitamin D deficiency.

**Results:**

Of 950 individuals analyzed, 29% were HIV-infected. The median age was 49 years; 65% were male, and 91% were black. The median vitamin D level was 13.5 ng/mL (IQR, 9.0–20.3); 74% were deficient (68% in HIV-infected vs. 76% in HIV-uninfected, p = 0.01). Non-black race, fall/winter season, multivitamin intake, higher serum albumin, HCV seropositivity and HIV-infection were associated with significantly lower odds of vitamin D deficiency.

**Conclusions:**

Vitamin D deficiency is prevalent among IDUs. Notably, HIV-infected IDUs were less likely to be vitamin D deficient. Higher vitamin D levels were associated with multivitamin intake and with higher albumin levels, suggesting that nutritional status contributes substantially to deficiency. The association between HCV serostatus and vitamin D level remains unclear. Further investigation is needed to define the clinical implications of the heavy burden of vitamin D deficiency in this high-risk, aging population with significant co-morbidities.

## Introduction

Vitamin D deficiency is common in the United States, affecting approximately 40% of American adults [1]. A sharp rise in the prevalence over the past 20 years has drawn significant attention to the health impacts of this widespread condition [2], including musculoskeletal disorders [3], [4], risk of falls [5], incident cardiovascular disease [6], cancer risk [7], and respiratory infections [8]–[10]. Despite heightened awareness regarding the adverse health outcomes associated with vitamin D deficiency, some populations remain at high risk for vitamin D deficiency and its complications.

Injection drug users (IDUs), in particular, often have poor nutritional status and limited, delayed access to healthcare [11]. As a result, this patient population suffers a disproportionate burden of vitamin D deficiency compared to other urban dwelling adults [12], [13]. Moreover, IDUs have increased risks for drug overdose, drug-related morbidity and mental health conditions which render the study of chronic conditions associated with vitamin D deficiency difficult [14]. Injection drug use increases the risk for a host of acute and chronic infectious and cardiopulmonary conditions that are associated with vitamin D deficiency [15]. Consequently, IDUs living in an urban environment represent a poorly studied population at high risk for vitamin D deficiency as well as its associated adverse effects.

The AIDS Linked to the IntraVenous Experience (ALIVE) study is comprised of current and former IDUs living in Baltimore, Maryland [16], providing the opportunity to determine the prevalence of vitamin D deficiency and identify potential risk factors in a large group of urban IDUs. Understanding these risk factors in this unique population is needed to improve targeted screening and intervention to prevent sequelae of this modifiable condition.

## Methods

### Ethics Statement

This study was approved by the Johns Hopkins School of Public Health Institutional Review Board and all patients provided written informed consent.

### Study Population

Since 1988, the ALIVE Study has prospectively followed a cohort of current and former IDUs living in Baltimore, Maryland [16]. As part of the ALIVE protocol, clinical, laboratory and behavioural data are collected semi-annually at study visits. Injection drug users followed between October 1, 2007 and May 30, 2008 for whom stored serum was available for vitamin D measurement were eligible for inclusion in our analysis. Of 1004 ALIVE participants evaluated during this time period, 951 had vitamin D levels measured cross-sectionally and comprised the population for this substudy.

### Data Collection

25(OH)-vitamin D levels were measured at the Tufts Medical Center Core Laboratory using radioimmunoassay (DiaSorin, Stillwater, Minnesota, USA). We excluded 1 participant whose value exceeded the maximum limit of detection. Vitamin D levels were analysed both continuously and categorically as deficient versus sufficient. Vitamin D deficiency was defined as a level less than 20 ng/mL [17].

T-cell subsets and HIV-1 RNA levels were measured at each study visit for HIV-infected participants (Roche Molecular Systems, Amplicor HIV-1 Monitor test version 1.5; Pleasanton, CA). Serum Hepatitis C Virus (HCV) antibody was measured at the first available visit after 2006, using an enzyme immunoassay (Ortho Diagnostics; Rochester, NY), while HCV RNA level was measured from plasma taken at or within 2 years of the vitamin D sample visit using Abbott real-time PCR (Abbott Molecular, Des Plaines, Illinois). Clinical and demographic data were obtained through study visit questionnaires; comorbidities were obtained through self-report and standardized medical record review. Race, income, insurance, healthcare resource utilization, drug use, and multivitamin intake were self-reported. Fall/winter season included September through February; spring included March through June. No levels were measured during summer months.

### Statistical Analyses

Descriptive characteristics of the study population are presented as frequencies, mean (standard deviation), or median (interquartile range [IQR]). Variables with skewed distributions such as HIV and HCV RNA were log_10_-transformed. Clinical and demographic characteristics were compared between deficient and sufficient adults using the *t* test, Wilcoxon rank-sum test or Pearson χ^2^, as appropriate. A two-sided p-value ≤ 0.05 was used to define statistical significance.

In our univariable and multivariable logistic regression models, we considered relevant demographic and clinical characteristics with a known association with vitamin D deficiency (age, race, body mass index [BMI] and season of measurement) [18]–[22]. The final multivariable model included covariates with p-values ≤ 0.05. Identical analyses were performed among HIV-infected participants only. Sensitivity analyses were performed in which associations were explored: 1) among black participants only; 2) with severe vitamin D deficiency (defined as <10 ng/mL); and 3) with HCV serostatus defined by both the antibody and RNA. All analyses were performed using Stata version 12.0 [23] and SAS version 9.0 [24].

## Results

### Participant Characteristics

The mean age of the 950 ALIVE participants was 49 years; 620 (65%) were male, 864 (91%) black and 278 (29%) HIV-infected (**Table 1**). Two-hundred and fifty-three (27%) participants self-reported multivitamin intake. Among HIV-infected participants, the median CD4+ cell count was 278 cells/mm^2^ (IQR: 166–174); 95 (34%) had a CD4 cell count <200 cells/mm^2^ and 105 (39%) had undetectable HIV viral load (<40 copies/mL). Among participants with detectable viral levels, the median HIV RNA level was 15,200 copies/mL (IQR: 1,560–59,450).

**Table 1:** Sociodemographic and Clinical Characteristics of Study Participants.

	VitD Sufficient ≥20 ng/mL		VitD Deficient <20 ng/mL		p-value
Number of participants	251		699		
Season					<0.001
Spring	22	(9)	195	(28)	
Summer	0	(0)	0	(0)	
Fall/Winter	229	(91)	504	(72)	
Mean age, years, mean(SD)	48.6	(8.9)	49.1	(7.6)	0.38
Black	212	(84)	652	(93)	<0.001
Non-Black	39	(15)	47	(7)	
Female	85	(34)	245	(35)	0.74
Homeless	31	(12)	83	(12)	0.82
Median annual income					0.99
No legal income	52	(21)	147	(22)	
<$5000	130	(52)	363	(53)	
≥$5000	62	(25)	170	(25)	
Medical insurance*	186	(75)	518	(74)	0.83
Outpatient medical visit*	171	(68)	420	(60)	0.03
Inpatient medical visit*	37	(15)	86	(12)	0.35
ER visit*	66	(26)	190	(27)	0.79
Injection drug use*	91	(36)	247	(35)	0.79
Multivitamin intake*	98	(39)	155	(22)	<0.001
Systolic BP, mmHg, mean(SD)	129	(25)	130	(22)	0.50
Diastolic BP, mmHg, mean(SD)	84	(15)	86	(14)	0.16
BMI, kg/m^2^, mean(SD)	26.4	(5.9)	26.7	(6.1)	0.51
Serum albumin ≤3.5 g/dL	20	(8)	85	(12)	0.07
History of diabetes	29	(12)	75	(11)	0.73
History of hypertension	118	(47)	370	(53)	0.10
HCV Antibody Seropositive	227	(91)	582	(83)	0.004
HCV RNA level, log_10_ copies/mL, mean (SD)†	6.4	(1.0)	6.4	(0.9)	0.48
HIV-infected	89	(35)	189	(27)	0.01
Prior AIDS‡	15	(17)	32	(17)	0.99
CD4+ cell count, cells/mm^3^ ‡	343	(198–584)	263	(148–449)	0.03
HIV RNA <40 copies/mL‡	46	(52)	64	(33)	0.003
HIV RNA level, log_10_ copies/mL, mean (SD) ‡	2.8	(1.4)	3.1	(1.4)	0.06
HAART receipt*	55	(63)	96	(52)	0.07

Values presented as n(%) or median (IQR) unless indicated otherwise.

* In the previous 6 months.

† n = 383

‡ Among participants with HIV.

Abbreviations: BMI, body mass index; BP, blood pressure; ER, emergency room; HAART, highly active antiretroviral therapy; HCV, hepatitis C virus; HIV, human immunodeficiency virus; IQR, interquartile range; kPA, kilopascal; RNA, ribonucleic acid; SD, standard deviation; UD, undetectable; VitD, 25(OH)-vitamin D; WBC, white blood cell.

### Vitamin D Deficiency among IDUs

The median vitamin D level was 13.5 ng/mL (IQR: 9.0–20.3) with 74% (n = 699) of the cohort having deficient levels. Vitamin D associations were explored with both a continuous (data not shown) and categorical vitamin D deficient variable (defined as 25(OH)-vitamin D <20 ng/mL). Vitamin D deficiency status did not vary by age, gender, or BMI (Table 1). Median vitamin D levels were similar among HIV-infected and HIV-uninfected subjects (13.8 vs. 13.4 pg/mL, p = 0.40). The prevalence of vitamin D deficiency, however, was lower among HIV-infected versus uninfected individuals (68% vs. 76%, respectively; p = 0.012). Among participants with HCV infection (n = 582), 72% were vitamin D deficient compared to 84% of HCV-uninfected participants (n = 117; p = 0.004).

In univariable analysis, black race was associated with a 2.55 increased odds of vitamin D deficiency (95%CI: 1.62, 4.01) (**Table 2**). In contrast, HCV antibody seropositivity (OR = 0.50; 95% CI: 0.31, 0.81) and HIV seropositivity (OR 0.67; 95% CI: 0.49, 0.92) were associated with lower odds of vitamin D deficiency as were season of blood draw, multivitamin intake and higher serum albumin. Participants seen in an outpatient clinic in the past 6 months also had lower odds of vitamin D deficiency (OR 0.71, 95% CI; 0.52, 0.96). However, active injection drug use, indicators of socioeconomic status (e.g., annual income, homelessness, and insurance status),history of incarceration and comorbid conditions were not significantly associated with vitamin D status.

**Table 2:** Association between Entire Cohort Characteristics and Vitamin D Deficiency (n = 950).

Predictor	Unadjusted OR (95% CI)		p-value	Adjusted OR (95% CI)		p-value
Age, per 10 years	1.08	(0.90, 1.30)	0.38	1.01	(0.82, 1.26)	0.89
Black Race	2.55	(1.62, 4.01)	<0.001	3.26	(1.89, 5.63)	<0.001
BMI, per 1 kg/m^2^	1.01	(0.98, 1.03)	0.51	1.00	(0.98, 1.03)	0.81
Fall/Winter Season*	0.25	(0.15, 0.40)	<0.001	0.23	(0.14, 0.37)	<0.001
Current Multivitamin Intake†	0.44	(0.32, 0.60)	<0.001	0.43	(0.31, 0.60)	<0.001
Serum albumin ≤3.5 g/dL	1.60	(0.95, 2.66)	0.07	1.82	(1.05, 3.17)	0.03
HCV Antibody Seropositive	0.50	(0.31, 0.81)	0.004	0.53	(0.31, 0.89)	0.02
HIV-infected	0.67	(0.49, 0.92)	0.01	0.70	(0.49, 1.00)	0.05
Any Outpatient Visit†	0.71	(0.52, 0.96)	0.03	0.83	(0.59, 1.17)	0.29

Models adjusted for other variables in table.

* As compared to spring season of measurement.

† In the previous 6 months.

Abbreviations: BMI, body mass index; CI, confidence interval; HCV, hepatitis C virus; HAART, highly active antiretroviral therapy; HIV, human immunodeficiency virus; OR, odds ratio.

In the multivariable model, black race remained strongly associated with higher odds of vitamin D deficiency (OR 3.26, 95% CI: 1.89, 5.63) (**Table 2**). In addition, hypoalbuminemia was associated with a nearly 2-fold greater odds of vitamin D deficiency (OR 1.82; 95% CI: 1.05, 3.17). HCV antibody seropositivity (OR 0.53; 95%CI: 0.31, 0.89) remained associated with reduced odds of vitamin D deficiency as did fall or winter timing of vitamin D measurements (OR 0.23; 95% CI: 0.14, 0.37) and multivitamin intake (OR 0.43; 95% CI: 0.31, 0.60). The association between HIV-infection and reduced odds of vitamin D deficiency was not substantially altered (OR 0.70; 95% CI: 0.49, 1.00).

### Factors Associated with Vitamin D Deficiency among HIV-infected IDUs

Univariable analyses among HIV-infected participants showed similar results as in the overall cohort analysis (**Table 3** **)**. Among HIV-infected IDUs, hypoalbuminemia remained strongly associated with vitamin D deficiency (OR 2.63; 95% CI: 1.33, 5.22). Conversely, risk of vitamin D deficiency was reduced during fall or winter measurement (as compared to spring; OR 0.22, 95% CI: 0.10, 0.52), among those reporting multivitamin intake (OR 0.50; 95% CI: 0.29, 0.83) and among those with undetectable HIV RNA (OR 0.43, 95% CI: 0.26, 0.73).

**Table 3:** Association between Patient Characteristics and Vitamin D Deficiency among HIV-Infected Participants (n = 278).

Predictor	Unadjusted OR (95% CI)		p-value	Adjusted OR (95% CI)		p-value
Age, per 10 years	0.70	(0.48, 1.03)	0.07	0.98	(0.93, 1.02)	0.35
Black Race	1.93	(0.68, 5.50)	0.22	3.26	(0.88, 12.00)	0.08
BMI, per 1 kg/m^2^	1.00	(0.96, 1.04)	0.90	1.00	(0.96, 1.05)	0.84
Fall/Winter Season*	0.22	(0.10, 0.52)	<0.001	0.20	(0.08, 0.48)	<0.001
Current Multivitamin Intake†	0.50	(0.29, 0.83)	0.01	0.59	(0.28, 0.87)	0.01
Serum albumin ≤3.5 g/dL	2.63	(1.33, 5.22)	0.01	2.04	(0.94, 4.45)	0.07
HCV Antibody Seropositive	0.29	(0.06, 1.29)	0.10	0.24	(0.05, 1.20)	0.08
Any Outpatient Visit†	0.77	(0.39, 1.50)	0.44	0.89	(0.42, 1.90)	0.77
HIV RNA level <40 copies/mL	0.43	(0.26, 0.73)	0.002	0.61	(0.30, 1.23)	0.17
Current HAART Use†	0.62	(0.37, 1.05)	0.07	1.00	(0.50, 1.96)	0.99

Models adjusted for other variables in table.

*As compared to spring season of measurement.

† In the previous 6 months.

Abbreviations: BMI, body mass index; CI, confidence interval; HCV, hepatitis C virus; HAART, highly active antiretroviral therapy; HIV, human immunodeficiency virus; OR, odds ratio.

In multivariable analyses of HIV-infected participants, fall or winter season of measurement and multivitamin intake remained associated with reduced odds of vitamin D deficiency (OR 0.20; 95% CI: 0.08, 0.48 and OR 0.59; 95% CI: 0.27, 0.87, respectively); however, hypoalbuminemia and HIV viral suppression no longer reached statistical significance.

### Sensitivity Analyses

Restriction of the analyses to black participants yielded similar results (data not shown). When examining severe vitamin D deficiency (defined as <10 ng/mL), race, season of blood draw, multivitamin intake, hypoalbuminemia and HCV antibody serostatus remained independently associated with vitamin D levels in additional to systolic blood pressure (OR 1.08 per 10 mm Hg higher; 95% CI: 1.01, 1.16); however, HIV serostatus no longer reached statistical significance (**Table S1**). The associations among HIV-infected participants were unchanged with the more stringent cut-off (data not shown).

To better understand the association between HCV and vitamin D status, we categorized participants into three categories: 1) HCV antibody negative; 2) HCV antibody positive with undetectable RNA; and 3) HCV antibody positive with detectable RNA. Compared with HCV antibody negative individuals, those who were HCV antibody positive and had either undetectable (OR 0.45; 95% CI: 0.23, 0.90) or detectable RNA (OR 0.50; 95% CI: 0.28, 0.90) remained at lower odds of vitamin D deficiency in adjusted models. Individuals who were HCV antibody positive, however, were more likely to have had an outpatient clinic visit within the preceding 6 months compared to those who were HCV antibody negative (64% vs. 48%, p = 0.001).

## Discussion

In our study, we found that three out of every four current or former IDUs were vitamin D deficient, similar to the high prevalence noted among blacks and HIV-infected adults in the United States [25], [26]. Significantly higher odds of vitamin D deficiency were observed with black race, lack of multivitamin use and hypoalbuminemia. Notably, HIV and HCV infected IDUs were less likely to be vitamin D deficient. To our knowledge, no prior studies have reported the prevalence or identified the correlates of vitamin D deficiency in an urban cohort of IDUs.

Vitamin D deficiency has become increasingly prevalent in the United States [2] and is associated with an array of poor health outcomes [6], [7], [27]–[35]. Repletion of vitamin D levels has been shown to attenuate some of these risks, specifically bone loss [36]–[39], falls risk [40]–[43], hypertension [44]–[46], insulin resistance [47], and mortality [27]. With increased risk for infectious and cardiopulmonary comorbidities [15] and delayed, limited access to healthcare [11], IDUs represent a population at high-risk for vitamin D deficiency and its sequelae. In addition, the safety and efficacy of vitamin D supplementation, along with bisphosphonate therapy, has recently been demonstrated among HIV-infected patients [48]. Establishing the prevalence and correlates of vitamin D deficiency among IDUs therefore would allow targeted screening and repletion.

Our cohort was predominantly black, which at least partially explains the elevated prevalence of vitamin D deficiency observed, as blacks are known to have lower levels of vitamin D compared to whites [25]. This racial difference may be explained by the reduction in cutaneous vitamin D photoproduction among blacks [49], [50], as well as decreased intake of dairy and dietary supplements [51].

Our findings support the seasonal variation often observed with vitamin D measurement [52], [53]. High levels of vitamin D in the late summer and early fall are typically contrasted with lower levels in the late winter and early spring [54]–[56] which correlates with our observed higher levels observed in the fall/winter season as compared to the spring. These findings highlight the importance of considering season when interpreting vitamin D results.

In addition to black race and season, we observed significantly greater odds of vitamin D deficiency associated with lack of multivitamin intake and with lower serum albumin; the latter, suggests that poor nutritional status may have contributed. Lower serum albumin is associated with inadequate protein and caloric intake, as well as inflammation, all of which may be present in a population of IDUs and contributing to vitamin D deficiency [57], [58]. Reduced vitamin D deficiency, as expected [59], was associated with multivitamin intake. Though multivitamins typically only contain supplemental doses of vitamin D, multivitamin use may indicate individuals who also take in a more balanced diet sufficient to prevent vitamin D deficiency.

Several prior studies have examined the association of HIV infection and vitamin D deficiency with conflicting results. While HIV infection has typically been associated with increased vitamin D deficiency [60], a few studies also found reduced odds of vitamin D deficiency similar to our findings [26], [61], [62]. HIV-infected patients with vitamin D deficiency had lower CD4+ cell counts and higher HIV RNA levels compared to sufficient participants suggesting that treatment for HIV also reduced risk of vitamin D deficiency, which has been noted previously [63]. However, multivariable analysis showed that recent outpatient clinic visits, HAART use and markers typically associated with inadequate HIV treatment including HIV RNA levels did not predict vitamin D deficiency. Measurement of free vitamin D, rather than total vitamin D, may have also played a role in these findings. Vitamin D circulates bound to plasma proteins including albumin and vitamin D binding protein; free vitamin D is the biologically active, and possibly more clinically relevant, measure of vitamin D.

We observed a similar “protective” association between HCV infection and vitamin D deficiency, in contrast to prior studies [64], [65]. HCV and HIV infection have been associated with discordant sex hormone levels due to elevated sex hormone binding proteins [66], [67]. A similar mechanism of increased vitamin D binding protein resulting in discordant total and free vitamin D levels may be contributing to our findings among these patients. HCV cirrhosis has been reported to be associated with reduced vitamin D binding protein levels [68]. Further investigation into free vitamin D levels is warranted in these populations, particularly in light of a recent study reporting that free vitamin D more strongly correlated with clinical outcomes than total vitamin D [69].

This study has limitations. Vitamin D measurements were performed only at a single time point, within a 9 month calendar period. We did not measure parathyroid hormone or vitamin D binding protein which would have enhanced our inferences. In this cross-sectional analysis, we are unable to infer causal associations with vitamin D deficiency. Our cohort largely consists of African American IDUs residing in an urban setting at relatively northern latitude which limits generalizability to other populations. Although nearly 30% of our participants were HIV-infected, 40% had undetectable HIV RNA at the time of investigation; therefore, our HIV findings may be applicable only to persons with a similar HIV treatment and response profile. Multivitamin intake is self-reported, is not characterized with regards to dose or duration of therapy, and may not reflect nutritional status, thereby limiting our inferences regarding its protective association with vitamin D deficiency. Despite this, ALIVE participants are similar to many other urban IDU populations in the United States. Further, our conclusions are strengthened by the large size and well-characterized nature of the cohort.

## Conclusions

We report a substantial prevalence of vitamin D deficiency among IDUs. In addition to expected associations of vitamin D deficiency with black race and season, we provide evidence that nutritional factors are important in this population. The reduced risk for vitamin D deficiency among participants with HIV or HCV infection evokes questions regarding the role of free vitamin D measurement in these unique populations. Future studies are needed to examine the adverse clinical outcomes associated with pronounced vitamin D deficiency and to evaluate whether routine screening for vitamin D deficiency followed by supplementation can ameliorate the potential health consequences among IDUs.

## Supporting Information

Table S1 **Association between Entire Cohort Characteristics and Vitamin D Deficiency defined as <10 ng/mL (n = 950).**
(DOC)
