# Supplementary material for: Risk Factors for Vitamin D Deficiency among HIV-Infected and Uninfected Injection Drug Users
Source: PLoS One. 2014 Apr 22;9(4):e95802. doi: 10.1371/journal.pone.0095802 (PMC3995810; doi:10.1371/journal.pone.0095802)
Supplement: Table S1 — Association between Entire Cohort Characteristics and Vitamin D Deficiency defined as <10 ng/mL (n = 950). (DOC) [file pone.0095802.s001.doc]

**SUPPLEMENTARY FILES**

**Risk Factors for Vitamin D Deficiency among HIV-infected and Uninfected Injection Drug Users**

Allison A. Lambert, MD, MHS; M. Bradley Drummond, MD, MHS; Shruti H. Mehta, MPH, PhD; Todd T. Brown, MD, PhD; Gregory M. Lucas, MD, PhD; Gregory D. Kirk, MD, PhD, MPH; Michelle M. Estrella, MD, MHS

| **Supplementary Table 1. Association between Entire Cohort Characteristics and Vitamin D Deficiency defined as <10 ng/mL (n=950)** | | | | | | | | |
| --- | --- | --- | --- | --- | --- | --- | --- | --- |
| **Predictor** | **Unadjusted OR**  **(95% CI)** | | **p-value** | | **Adjusted OR**  **(95% CI)** | | **p-value** | |
| Age, per 10 years | 1.06 | (0.89, 1.27) | | 0.48 | 0.97 | (0.78, 1.22) | | 0.83 |
| Black Race | 3.55 | (1.80, 6.97) | | <0.001 | 3.98 | (1.90, 8.31) | | <0.001 |
| Systolic blood pressure, per 10 mm Hg | 1.08 | (1.02, 1.15) | | 0.01 | 1.08 | (1.01, 1.16) | | 0.03 |
| BMI, per 1 kg/m^2^ | 0.55 | (0.38, 0.80) | | 0.22 | 0.97 | (0.95, 1.00) | | 0.05 |
| Fall/Winter Season* | 0.36 | (0.26, 0.49) | | <0.001 | 0.32 | (0.23, 0.45) | | <0.001 |
| Current Multivitamin intake† | 0.55 | (0.39, 0.77) | | <0.001 | 0.50 | (0.35, 0.72) | | <0.001 |
| Serum albumin ≤3.5 g/dL | 2.58 | (1.71, 3.89) | | <0.001 | 2.59 | (1.63, 4.09) | | <0.001 |
| HCV Antibody Seropositive | 0.50 | (0.31, 0.81) | | 0.002 | 0.43 | (0.28, 0.66) | | <0.001 |
| HIV-infected | 1.17 | (0.87, 1.58) | | 0.03 | 1.18 | (0.82, 1.70) | | 0.37 |
| Any Outpatient Visit† | 0.94 | (0.71, 1.25) | | 0.67 | 1.02 | (0.74, 1.41) | | 0.90 |
| Models adjusted for other variables in table.  * As compared to spring season of measurement.  † In the previous 6 months.  Abbreviations: OR, odds ratio; CI, confidence interval; BMI, body mass index; HCV, hepatitis C virus; HAART, highly active antiretroviral therapy; HIV, human immunodeficiency virus; OR, odds ratio. | | | | | | | | |
